# Costs structure of the inpatient ischemic stroke treatment using an exact costing method

**DOI:** 10.1016/j.heliyon.2020.e04264

**Published:** 2020-06-23

**Authors:** Anne Puumalainen, Outi Elonheimo, Mats Brommels

**Affiliations:** a Department of Public Health, University of Helsinki, Kajavankatu 2C 79, 04230, Kerava, Finland; b Network of Academic Health Centres and Department of General Practice and Primary Health Care, University of Helsinki, Helsinki, Finland; c Department of Learning, Informatics, Management and Ethics, Karolinska Institutet, Stockholm, Sweden

**Keywords:** Economics, Neurology, Ischemic stroke, Inpatient episode, Costs, Cost accounting methods, Activity-based costing

## Abstract

**Objectives:**

Most stroke care expenses are inhospital costs. Given the previously reported inaccuracy of conventional costing, the purpose of this study was to provide an accurate analysis of inpatient costs of stroke care in an acute care hospital.

**Materials and methods:**

We used activity-based costing (ABC) for calculating the costs of ischemic stroke patients. We collected the activity data at the Helsinki University Central Hospital. Persons involved in patient care logged their activities on survey forms for one week. The costs of activities were calculated based on information about salaries, material prices, and other costs obtained from hospital accounting data. We calculated costs per inpatient days and episodes, analyzed cost structure, made a distinction in cost for stroke subtypes according to the Oxford and TOAST classification schemes, and compared cost per inpatient episode with the diagnoses-related group (DRG) -price of the hospital.

**Results:**

The sample comprised 196 inpatient days of 41 patients. By using the ABC, the mean and median costs of an inpatient day were 346 € and 268 €, and of an inpatient episode 3322 € and 2573 €, respectively. Average costs differed considerably by stroke subtype. The first inpatient day was the most expensive. Working time costs comprised 63% of the average inpatient day cost, with nursing constituting the largest proportion. The mean cost of an inpatient episode was 21% lower with ABC than with DRG pricing.

**Conclusion:**

We demonstrate that there are differences in cost estimates depending on the methods used. ABC revealed differences among patients having the same diagnosis. The cost of an episode was lower than the DRG price of the hospital. Choosing an optimal costing method is essential for both reimbursements of hospitals and health policy decision-making.

## 1 Introduction

The stroke burden continues to increase [1]. In Finland, the mean one-year costs after an ischemic stroke are approximately 21 300 €, and the average stroke-related lifetime healthcare costs 60 000 €. The sum annually spent in the treatment of stroke patients in Finland is 7% of total healthcare costs, and stroke is one of the most significant causes of hospitalization [2, 3, 4, 5, 6]. Institutional care constitutes the largest proportion of costs [4, 5, 7, 8], and length of stay in hospital is the most critical determinant of high total costs [6, 9]. Accordingly, correct calculation of inpatient treatment costs is crucial [10].

Traditionally, total hospital costs have been calculated based on the sum of the values of inpatient days and outpatient visits. Unit costs have been computed generally at the level of the department as specialty averages, rather than at a specific patient level [10, 11]. Currently, the method most used for standard pricing of hospital care is diagnosis-related groups (DRG) [12]. DRG is a system classifying in-hospital patient cases into clinically meaningful categories with a similar use of resources. Its objective is to relate patients to the resources needed to treat them [12, 13], and DRG cost calculations are based on average resource consumption per diagnosis group. The method has been criticized for reflecting insufficiently patient-level treatment costs [14]. The values of diagnostic and therapeutic procedures, as well as expensive drugs, are specified by category, but nursing care, typically representing about 50% of the total costs, is handled as a unit overhead, equal for all inpatients [12].

Individual patients show different patterns of service utilization and resource use, and the real costs vary depending on the severity of the illness and the intensity of the treatment [15, 16, 17]. Royle et al. [18] reported that DRGs fail to predict the length of stay accurately, and according to a Dutch study [19], DRG costing explained only 34 % of the variation of the costs of stroke care. This proportion increased when the functional status and comorbidities of patients were taken into account in the calculations. Consequently, the average DRG costing poorly reflected the actual treatment costs of a specific patient, and this is also the case in Finland with the DRG group for stroke (NordDRG 014) [6]. Moreover, since the accounting systems of Finnish hospitals vary, the introduction of DRGs has been unsuccessful in making comparisons of hospital treatment costs valid [20].

In the traditional costing methods —known top-down costing — the organization's overhead costs are first allocated downwards to the departments and then to individual services, and only the costs that can be allocated to the product directly are identified as direct costs [21]. An alternative to conventional accounting methods is the bottom-up microcosting methods, argued to produce more accurate information than traditional costing methods regarding the costs of both products and services [22]. The most common bottom-up method is activity-based costing (ABC), where the costs are analyzed from a service process perspective. In ABC, processes are defined as a sequential set of activities needed to establish the service or product. The rationale is that by allocating costs to activities instead of directly to products, a clear relationship can be established between sources of activity demand and costs. The two-stage ABC process of cost allocation captures more costs as direct, thus typically having a lower proportion of indirect costs (overhead) than conventional costing [14, 17, 23, 24, 25].

In ABC, a health care service product, such as an inpatient day or inpatient episode, is formed by a series of care activities, and it requires an accurate estimation of time spent in various activities and other resources consumed by these activities. The cost of the service is then the sum of the costs of the resources used by all activities forming the service. Since health care is labor-intensive, the most critical cost driver of the activities is the working time used. All other resources, such as materials and services, are also allocated to the activities as accurately as is feasible, instead of allocating them directly to the service products [22, 26].

ABC became popular in the early 1980s, mainly because of growing dissatisfaction with traditional ways of allocating costs. The method was, however, tedious and required the collection of additional activity data. Consequently, only limited inpatient ABC applications were reported. In recent years, an updated and less labor-intensive version, time-driven activity-based costing, has been introduced, and ABC has again become a topical tool for improving cost measurement and management in healthcare organizations [11, 23, 27, 28]. With ABC, it is possible to make a cost differentiation between different kinds of patients based on their care processes [24].

## 2 Purpose

Given the reported inaccuracy of conventional costing, the purpose of this study is to provide with activity-based costing a more accurate analysis of inpatient costs of ischemic stroke in an acute care hospital. First, calculations include both the total costs of an inpatient episode and the costs per inpatient days, and we also evaluated the cost structure. Second, we calculated the costs separately for the subtypes of ischemic stroke, according to the Oxford [29] and TOAST [30] classification schemes. Finally, we compared the cost per stroke inpatient episode calculated with ABC with the DRG price of hospital.

## 3 Materials and methods

We collected the data at the Department of Neurology, Helsinki University Central Hospital, Finland in 2003. All acute neurological patients are treated in the department. The majority of the patient are ischemic stroke patients, and one of the four neurological wards is an acute stroke unit with beds for patients requiring intensive care. The stroke unit and two other neurological units carried out the activity survey for ischemic stroke patients for one week. All persons involved in patient care logged their activities on patient-specific survey forms. Each occupational group had its own form, including 15–25 key activities such as the intake or discharge of a patient, the medical round (doctors), assisting the patient in moving, eating or washing (nurses), patient examination, and various therapies (therapists). A neurologist divided the patients into subgroups by using the five-part Oxford-classification describing the location and extent of the infarction [29] as well as the five-part TOAST-classification describing the etiology of the disease [30].

When performing ABC, it is essential to describe the care process in sequential steps and to identify and define the activities that form the product. In this study, the “product” was the inpatient episode of care, covering the hospital stay from admission to discharge. The description of the product and activities was made together with the management and staff of the department. Each occupational group identified their patient care activities, covering both direct and indirect care, and combined the lesser activities into larger activity groups to keep the list of activities and the corresponding measurements manageable. We identified all of the resources consumed by the activities. Because the working time of professionals engaged in an activity is a substantial cost driver in inpatient care, we estimated the average working time per activity in a nominal group process, which involves staff representatives. Previously performed working time measurements at the department were used to guide the estimations.

Information on salaries, material and medication prices, and other costs was obtained from hospital accounting data. We calculated the cost per minute of working time for each occupational group from salary statistics. The working time cost of activity was the time needed for the activity multiplied by the cost per minute across occupational groups. The total cost of activity also included the costs of materials or services (such as typing or computer services) needed for this particular activity. The costs of any laboratory and radiography examinations, traced by the patient in the accounting system of the hospital, were added to the cost of the inpatient day in question.

Finally, we surveyed the costs of medication and intravenous fluids for a randomly selected sample of the patients and added the average (standard) medication cost to the cost of the inpatient day. We allocated the basic service (“hotel”) costs and salary costs of staff not directly involved in patient care as an average standard cost to inpatient days, but we omitted the capital costs or charges, rents, and costs of the hospital administration from the calculations. The process steps are described in Table 1.

**Table 1:** ABC -process steps in our study.

1. Identification of the "product."
2. Identification and definition of the activities that form the product.
3. Identification of all resources consumed by the activities.
4. Calculation of the cost/activity.
5. Registration of patient-level activities.
6. Calculation of the cost/inpatient day.
7. Calculation of the cost/inpatient episode.

In this paper, we report the costs per inpatient day and inpatient episode, both as means with standard deviations and as medians. For calculating the average cost of an inpatient episode, we multiplied the cost per inpatient day (both mean and median) with patients’ average length of stay. The DRG price of stroke was obtained from the financial administration of the hospital. DRG prices are calculated each year and are based on the trimmed cost averages of inpatient episodes. We used the DRG price of the same year as our data was collected and compared the mean cost of inpatient episodes calculated by ABC with the DRG price. As the study intention was cost accounting, we collected limited underlying factors: patient age, sex, Oxford- and TOAST-classifications, thrombolysis, and the care unit. Multivariable linear regression analysis was used to examine the effects of all the collected patient- or treatment-specific factors on the difference between the ABC calculated costs of the inpatient episode and the DRG -price. A two-sided value of P < 0.05 was considered to indicate statistical significance. Statistical analyses were carried out using SPSS Statistics 23 (SPSS, Inc., Chicago, IL, USA). The medical ethical committee of the ophthalmology, otorhinolaryngology, neurology and neurosurgery of Helsinki University Central Hospital and the steering committee of the Department of Neurology approved the study protocol.

## 4 Results

The study sample consisted of 196 ischemic stroke care days of 41 different patients, with nine patients requiring intensive care. The mean age of patients was 69.7 years (SD 9.3), and 20 (49%) of the patients were males and 21 (51%) females. Costs of medication and intravenous fluids were surveyed for 19 patients (mean age 64.7 years, SD 13.6) for 84 inpatient days. The distribution of all patients and inpatient days by Oxford and TOAST stroke subtypes is provided in Table 2.

**Table 2:** The distribution of patients and inpatient days by Oxford and TOAST stroke subtypes.

Stroke subtype classification	Patients (n = 41)		Inpatient days (n = 196)	
	FRQ∗	%	FRQ∗	%
Oxford classification				
Total anterior circulation infarct	7	17.1	32	16.3
Partial anterior circulation infarct	18	43.9	86	43.9
Posterior circulation infarct	12	29.3	58	29.6
Lacunar circulation infarct	4	9.8	20	10.2
TOAST classification				
Large vessel atherosclerosis	11	26.8	39	19.9
Cardioembolic	18	43.9	95	48.5
Small vessel occlusion	5	12.2	25	12.8
Stroke of other determined etiology	1	2.4	5	2.6
Stroke of undetermined etiology	6	14.6	32	16.3

∗ FRQ = frequency.

The ABC- calculated mean cost of an inpatient day was 346 €, and the median cost was 268 €. Multiplying these costs with the average length of stay (9.6 days), the mean and median cost of an inpatient episode was 3322 € and 2573 €, respectively. The mean cost of an inpatient episode was 21% lower than the price (4230 €) of the NordDRG group 014, which includes the ischemic stroke. According to the results of the linear regression, only the unit predicted the cost difference (Table 3). The stroke unit has the smallest difference between the ABC- calculated cost of the care episode and the DRG price.

**Table 3:** Effects of various factors on the cost difference between ABC-calculated cost of care episode and the DRG price of the hospital analysed by multivariable linear regression.

Variable	Unstandardized Coefficients		Standardized Coefficient	Significance	Confidence intervals
	B	Standard error	Beta	Sig.	95% CI for B
(Constant)	3876,97	2719,36		0,16	-1643,61–9397,56
Age	-6,97	27,24	-0,04	0,80	-62,26–48,33
Sex	-939,42	522,60	-0,26	0,08	-2000,36–121,51
Oxford- classification∗	426,12	292,46	0,21	0,15	-167,60–1019,84
Toast-classification∗∗	80,12	203,21	0,06	0,70	-332,43–492,66
Care unit	-864,14	333,54	-0,38	0,01	-1541,25 - -187,03

∗ Classification describing the location and extent of the infarction.

∗∗ Classification describing the etiology of the disease.

The mean and median cost of an inpatient day and inpatient episode differed considerably by stroke subtype, as shown in Table 4. The inpatient episode of total anterior circulation infarct costs twice as much as that of lacunar infarct. Among TOAST classified groups, large artery atherosclerosis was the most and small vessel occlusion the least costly subtype. There were differences in the costs of care units as well. The costs were the highest in the stroke unit, especially in stroke intensive care.

**Table 4:** The average inpatient day and total inpatient care episode cost by ischemic stroke subtypes and care unit.

	Inpatient day costs €		Inpatient care episode costs €	
	Mean (SD)	Median	Mean	Median
Oxford-classification∗				
Total anterior circulation infarct	434 (297)	317	4170	3049
Partial anterior circulation infarct	345 (211)	272	3312	2620
Posterior circulation infarct	345 (272)	278	3312	2673
Lacunar circulation infarct	207 (162)	136	1991	1311
TOAST-classification∗∗				
Large vessel atherosclerosis	432 (270)	391	4142	3751
Cardioembolism	327 (207)	267	3136	2566
Small vessel occlusion	265 (260)	145	2542	1389
Stroke of other determined etiology	442 (555)	154	4241	1482
Stroke of undetermined etiology	345 (235)	262	3312	2512
Care unit				
Neurological unit A	145 (30)	139	1394	1339
Neurological unit B	308 (122)	269	2956	2583
Stroke unit	336 (108)	325	3227	3124
Stroke unit intensive care	676 (262)	624	6490	5987
Total	346 (247)	268	3318	2568

∗ Classification describing the location and extent of the infarction.

∗∗ Classification describing the etiology of the disease.

**Figure 1:**
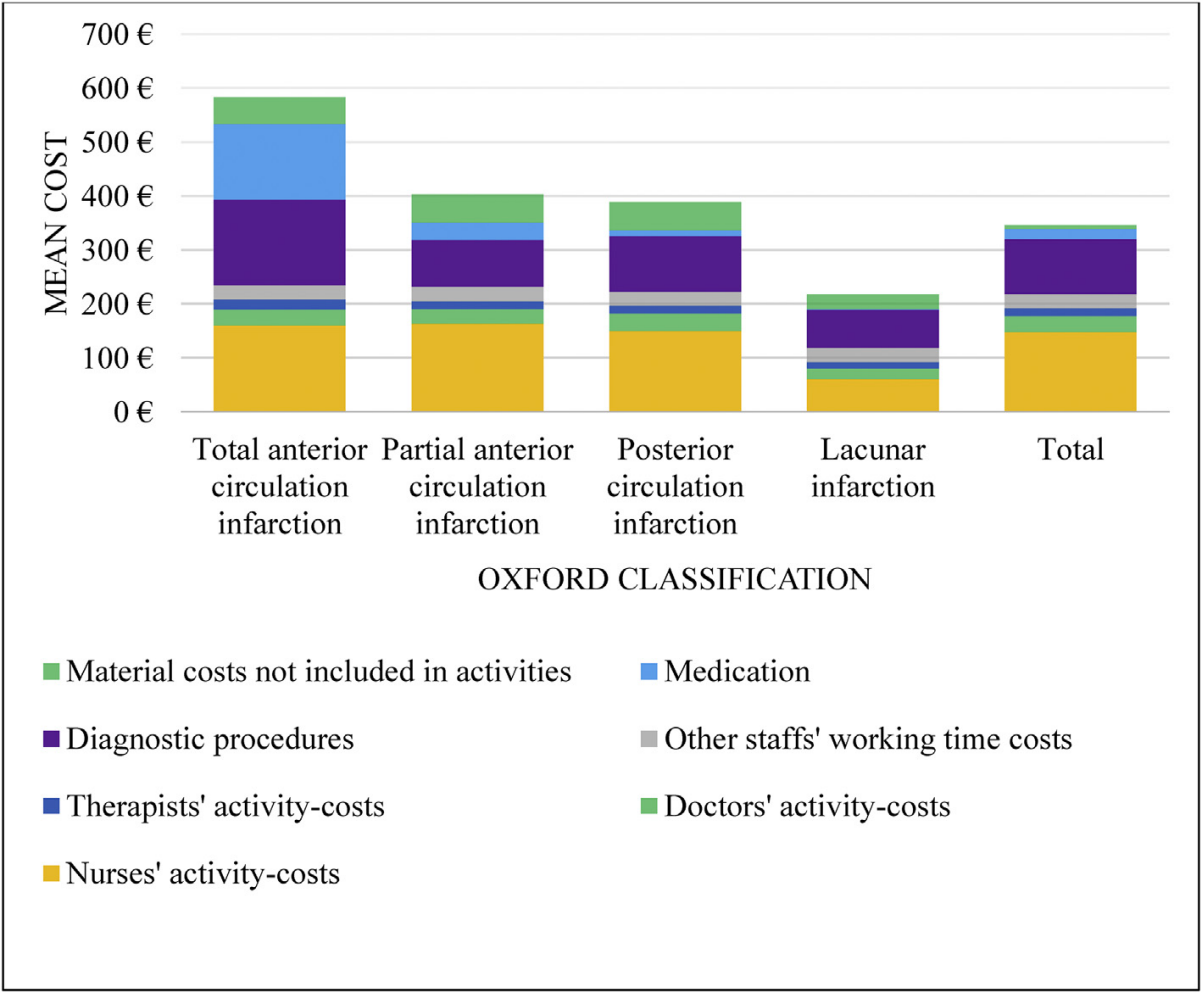
Structure of average inpatient day costs (€) of ischemic stroke treatment by Oxford classification.

**Figure 2:**
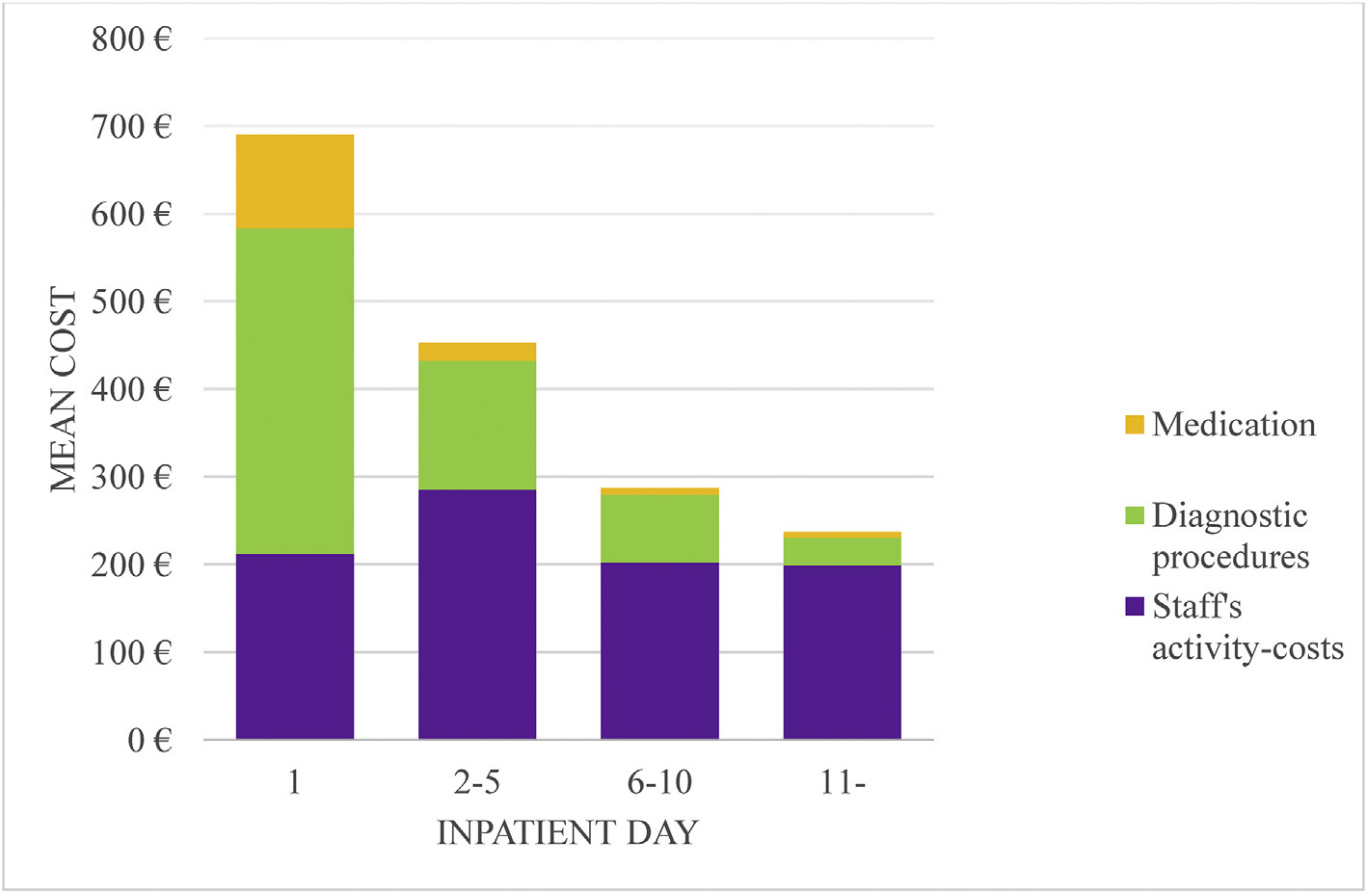
Structure of average inpatient day costs (€) of ischemic stroke treatment by inpatient day.

Working time costs comprised 63% of the average inpatient day cost of all patients, and nursing constituted the most significant portion of working time costs (Figure 1.). Diagnostic procedures and medication together accounted for approximately 35% of inpatient day costs. However, total anterior circulation infarct differed from the other subtypes such that diagnostic procedures and medication constituted 44% of day costs. As shown in Figure 2, the first inpatient day was the most expensive. The mean day costs decreased to half of the first day's costs during the course of a week. Diagnostic tests and expensive medicines mainly caused the high costs of the first care day.

## 5 Discussion

In the stroke patients’ inpatient hospital treatment, ABC provides more detailed information on the cost structure of the care than the earlier used methods. Moreover, it revealed differences among patients having the same diagnosis, an issue taken into consideration by only a few cost accounting methods. The location and type of infarction, as well as the etiological factors, influenced the treatment costs; both the cost of an inpatient episode and the daily costs vary between infarction subtypes. Only a small number of previous studies [9, 31] have revealed differences in expenses between ischemic stroke subtypes. Compared with the conventional costing methods, such as DRG pricing, the cost profile obtained with ABC is more precise.

Among other objectives, the DRG was developed to improve the hospital cost accounting. It relates patients to the resources needed to treat them and calculates the treatment cost of a group based on average resource consumption. The EuroDRG group [6], however, suggest that DRG grouping should include factors determining the patient's condition in more detail to better explain the cost variation. According to our study, the ischemic stroke subtype could be one of those factors, since resources are used in different amounts to treat patients having a different kind of infarct. Consequently, the costs of care are different.

The average prices of DRG groups have also been criticized [14] for reflecting insufficiently to the treatment cost variation because calculations include no care activities. As stroke treatment is especially labor-intensive, labor costs constitute the most substantial portion of total costs. According to our results, labor costs measured by activities represent over 60% of average inpatient day costs, with nursing being the most significant single cost component. Although Helsinki University Central Hospital has the most advanced hospital cost accounting system in Finland, DRG pricing handles the labor costs as an overhead, equal for all inpatients in the department or unit [12].

The cost of an inpatient episode that we calculated with ABC was 21% lower than the DRG price of the hospital. We used a specific diagnostic grouping as a product definition, and the lower cost can probably be partly explained by the NordDRG group 014, including also other, more expensive [2] cerebrovascular diseases apart from ischemic stroke. Other studies [14, 24] have found that the ABC calculated costs are either higher or lower than the corresponding DRG or other product prices. They explain the difference by the fact that ABC allocates a larger proportion of the total costs and therefore results in a considerably lower proportion of unallocated (overhead) costs, resulting in greater accuracy of the product unit cost.

The main limitation of this paper is that the data used were collected in the early 2000s. Since then, intravenous thrombolysis and, most recently, endovascular treatments have developed substantially. These two advanced therapies have been shown to lead to reduced mortality and improved functional outcomes of ischemic stroke patients [32, 33], which can shorten the length of hospital stay [34, 35]. Despite higher costs in the acute phase, thrombolysis and endovascular thrombectomy have been established to be cost-effective [36, 37]. Intravenous thrombolysis and endovascular therapies are, however, unsuitable for many stroke patients; a recent survey showed that in Finland, 12.6% of stroke patients received intravenous and only 3.6% endovascular treatment [38]. Most patients continue to receive usual medical care and rehabilitation. In the future, as stroke therapies evolve, there is an increasing need for the use of accurate costing methods.

Since ABC requires that the staff caring for patients is involved in the collection of data needed for the calculations, the results depend on how accurately they record the activity data. We cannot exclude the effects of a lack of logging on our predictions. However, we have no indications that recording irregularities occur unevenly across diagnostic subgroups causing systematic errors. While this kind of approach works in a limited setting, difficulties arise when applying it on a large scale on an ongoing basis. Modified methods, such as time-driven ABC are probably more practical for assigning costs of different medical conditions both accurately and relatively effortlessly [23, 39]. Computer-based solutions could also be useful for collecting the activity data on a routine basis.

Since the length of hospital care is the most significant predictor of in-hospital and total stroke care costs [6, 40, 41], the accounting method used should provide specific information on overall care costs, instead of the expenses of single procedures. Activity-based costing was shown to be suitable for calculating the costs of stroke patient care and revealing differences between subgroups of stroke. According to our regression analysis, the care unit predicted the difference between ABC-calculated costs and the DRG price of the hospital. The difference between units could be explained by the patient casemix; the stroke unit takes care of the most severe cases. However, the mean unit cost of an episode was considerably lower than the corresponding DRG price used for reimbursement of stroke patient care. Hospitals that receive funding based on DRGs could thus be overcompensated.

In economic studies, faulty assumptions of hospital care costs lead to incorrect results, which have effects on health policy decision-making. The value of acute hospital care of stroke patients as a proportion of total costs may be overestimated, while the costs of rehabilitation and long-term care are underestimated. Overly high estimations of the costs of the acute hospital care may lead to less emphasis on securing a well-functioning emergency service and stroke units, with the risks of human suffering and financial losses. Assuming these faulty assumptions lead to low priority given to sufficient care capacity in acute care, it can cause higher total costs over the entire episode of care. An accurate costing method in hospitals is crucial, and according to our results, there are differences in cost estimates, depending on the method used.

## Declarations

### Author contribution statement

Anne Puumalainen: Conceived and designed the experiments; Performed the experiments; Analyzed and interpreted the data; Contributed reagents, materials, analysis tools or data; Wrote the paper.

Outi Elonheimo: Conceived and designed the experiments; Analyzed and interpreted the data; Wrote the paper.

Mats Brommels: Conceived and designed the experiments; Wrote the paper.

### Funding statement

This work was supported by Helsinki University Central Hospital EVO Funding and 10.13039/100010114Yrjö Jahnsson Foundation.

### Competing interest statement

The authors declare no conflict of interest.

### Additional information

No additional information is available for this paper.
